# The inflammatory biomarkers profile of hospitalized patients with COVID-19 and its association with patient’s outcome: A single centered study

**DOI:** 10.1371/journal.pone.0260537

**Published:** 2021-12-02

**Authors:** Ibrahim Y. Hachim, Mahmood Y. Hachim, Haifa Hannawi, Kashif Bin Naeem, Abdulla Salah, Suad Hannawi

**Affiliations:** 1 Sharjah Institute for Medical Research, University of Sharjah, Sharjah, United Arab Emirates; 2 College of Medicine, University of Sharjah, Sharjah, United Arab Emirates; 3 College of Medicine, Mohammed bin Rashid University of Medicine and Health Sciences, Dubai, United Arab Emirates; 4 Mohammed bin Rashid University of Medicine and Health Sciences, Dubai, United Arab Emirates; 5 Ministry of Health and Prevention (MOHAP), Dubai, UAE; 6 Gulf Medical University, Ajman, United Arab Emirates; Heidelberg University Hospital, GERMANY

## Abstract

Several reports highlighted the central role of inflammation in the pathogenesis of corona virus disease-19 (COVID-19) disease. Also, the hyper-inflammatory response that is triggered by severe acute respiratory syndrom-Covid-2 (SARS-CoV-2) infection was believed to play an essential role in disease severity and adverse clinical course. For that reason, the classical inflammatory markers were proposed as a possible indicator for COVID-19 severity. However, an extensive analysis of the predictive value of inflammatory biomarkers in large patients’ cohorts is still limited and critically needed. In this study we investigated the predictive value of the classical inflammatory biomarkers in a patient cohort consists of 541 COVID-19 patients admitted to Al Kuwait Hospital, Dubai, UAE. A detailed analysis of the association between the essential inflammatory markers and clinical characteristics as well as clinical outcome of the patients were made. In addition, the correlation between those markers and a wide range of laboratory biomarkers and incidence of acute organs injury were investigated. Our results showed a significant elevation of many inflammatory markers including white cell count (WBC) count, neutrophils count, C-reactive protein (CRP), D-Dimer, ferritin, procalcitonin (PCT), and lactate dehydrogenase (LDH) levels in patients with more severe illness. Also, our results highlighted that higher levels of those markers can predict worse patient outcome including the need of ventilation, intensive care unit (ICU) admission, multiple organs dysfunction as well as death. In addition, Our results showed that the presence of lymphopenia and lower absolute lymphocyte count (ALC) at the time of admission were associated with severe to critical COVID-19 illness (P<0.0001), presence of acute respiratory distress syndrome (ARDS) (P<0.0001) and the need for ventilation and ICU admission., Moreover, our results showed a strong association between lower ALC count and multiple organs dysfunction and patient’s death (P<0.0001). In conclusion, our results highlighted the possible use of classical inflammatory biomarkers at time of admission as a potential predictive marker for more severe clinical course in COVID-19 patients that might need more aggressive therapeutic approach including the need of ventilators and ICU admission. The presence of such predictive markers might improve patient’s stratification and help in the direction of the available resources to patients in need, which in turn help in improving our response to the disease pandemic.

## Introduction

While approximately 80% of corona virus disease-19 (COVID-19) patients usually suffer from mild/moderate symptoms that needs minimal medical intervention, around 20% of COVID-19 patient progress to more severe form of the disease that require hospitalization with the need for ventilation and intensive care unit (ICU) admission [1–3]. For that reason, enormous efforts were made to improve our understanding of COVID-19 pathogenesis as well as risk factors involved in adverse clinical outcome of the disease in some patients. Indeed, this might lead to the discovery of predictive markers that can identify patients with severe/critical disease who need more aggressive medical interference [4–11]. Indeed, this step is essential for the early management of such patients to improve their outcome and to facilitate the optimal use of the available medical infrastructures and resources [12].

Many reports highlighted that inflammation in addition to the direct viral damage are essential mechanisms that might contribute to COVID-19 severity [13, 14]. Due to the critical role of inflammation in the pathogenesis and progression of COVID-19 disease, as well as the increment of proinflammatory cytokines in the serum of COVID-19 patients, many attempts were made to investigate the role of inflammatory markers in predicting COVID-19 disease’s severity as well as patient outcome [15–17]. Indeed, higher levels of inflammatory markers including C-reactive protein (CRP), neutrophil-to-lymphocyte ratio as well as various inflammatory cytokines and chemokines were found to be linked to more severe clinical course in COVID-19 patients [17–20].

Many of those reports focused only on the patient’s clinical characteristics and investigated only selected biomarkers [9]. The aim of our study is to analyze the association between inflammatory markers and features of disease progression and outcome in COVID-19 patients. This includes the severity of the disease, ICU admission, mortality as well as laboratory indicators. We achieve our aim through investigated those markers in a large patient’s cohort consisting of 541 COVID-19 patients. We also investigated the association between those markers as well as a wide range of biomarkers and evidence of organ injuries.

## Material and method

### Patients cohort and COVID19 severity indicators

A 541 patients diagnosed with COVID-19 infection, who were admitted to Al Kuwait Hospital, Dubai, United Arab Emirates (UAE) were included in our study. The cases were confirmed to be COVID-19 using the polymerase chain reaction (PCR) from nasal swab sample. The Sacace Real Time Reverse Transcription Polymerase Chain Reaction (rRT-PCR) test was used in our laboratory.

Our study was approved by the Ministry of Health and Prevention (MOHAP) Research Ethics Committee (MOHAP/DXB-REC/MMM/NO.44/2020). All the clinical, laboratory and demographic, clinical and laboratory characteristics of patients were retrieved from the electronic file system. The COVID-19 disease severity was classified according to the following criteria: 1—mild to moderate COVID-19 disease, if there was no or only evidence of mild pneumonia based on chest radiography or chest computed tomography (CT) findings; 2—severe COVID-19 disease, if there was dyspnea, respiratory rate ≥ 30/minute, blood oxygen saturation ≤ 93%, PaO2/FiO2 ratio < 300, and/or lung infiltrates >50% within 24–48 hours; and 3—critical COVID-19 infection, if there was respiratory failure, septic shock, and/or multiple organ dysfunction/failure. Estimated glomerular filtration rate (eGFR) was calculated according to the modification of diet in renal disease (MDRD) formula; 186 × (SCr mg/dl)-1.154 x (age)-0203 × 0.742 [if Female] x 1.212 [if Black], to calculate the eGFR for all participants. The laboratory profile includes hematological profile that involves hemoglobin (Hb) (normal reference range;: female: 13.5–17.5 g/dl, male: 12.0–15.5 g/dl,) white cell count (WCC), normal reference range: 4,000–11,000x10(3)/mcl), neutrophil count (normal reference range: 1.5–4 x10(3)/mcl), absolute lymphocyte count (normal reference range: 1,000 and 4,800 x10(3)/mcl) (lymphopenia was defined if lymphocyte count was < 1 x103/mcL), platelet count (normal reference range: 150,000–400,000 x10(3)/mcl). In addition, our results include classical inflammatory markers like CRP (normal reference range: 0.0–5.0 mg/liter), ferritin (normal reference range: males: 26–388 ng/mL, and female: 8–252 ng/mL), PCT (normal reference range < 0.1 ng/mL). In addition, we also investigated the levels of lactate dehydrogenase (LDH) (normal reference range: Male: 85–227 U/liter, and Female: 81–234 U/liter), which is considered as an indicator of tissue damage and recently found to be a risk factor for ARDS in CoVID-19 patients [21]. Our profile also includes liver function test that involves alanine transaminase (ALT), aspartate aminotransferase (AST), albumin, bilirubin, and alkaline phosphatase level. Renal function tests involving blood urea, creatinine and electrolytes profile including sodium (Na) and potassium (K) levels as well as coagulation profile, involving international normalized ratio (INR) and D-dimer.

The presence of organ failure or injury was considered according to a criteria previously described [8]: Acute cardiac injury was defined as the presence of at least one documented elevated high-sensitivity troponin-I. Acute kidney injury was defined as patient having a raise in the serum creatinine of ≥26.5 μmol/liter within 48 h.; or increase in serum creatinine ≥1.5 times than the baseline that occurred within the preceding 7 days; or if there were reduction in the urine volume <0.5 ml/kg/hr. for 6 consecutive hours. Acute liver injury was defined as patient having high ALT and/or high AST by more than 5 times the upper limit of normal range. In addition to the definition of ARDS which was made using Berlin definition [22].

## Statistical analysis

Patient characteristics and laboratory profile were summarized and tabulated using the standard descriptive statistics. All our variables were continuous variables. For that reason, data were tabulated and presented as mean ± SD. SPSS 21.0 (BM Corporation, Armonk, NY) software was used for the statistical analysis. Pearson correlation was used to assess the correlation between inflammatory markers and different laboratory indicators. A p-value of <0.05 was used as a cut-off value to differentiate between significant or non-significant differences.

## Results

The demographic, clinical as well as the laboratory profile of our patient’s cohort that consist of 541 patients is demonstrated in Table 1.

**Table 1:** The demographic and clinical as well as the laboratory indicators of 541 symptomatic COVID-1 patients’ cohort.

Variables	No.(%)
**Age (mean± SD)**	48.64±14.78
**Gender (No., %)**	
Male	416 (76.89%)
Female	125 (23.10%)
**BMI**	28.57 ±5.80
**Time from symptom onset to admission, mean±SD, days**	5.7±3.0
**Disease severity**	
Mild-moderate	189(34.93%)
Sever	203(37.52%)
Critical	149(27.54%)
**ICU admission**	153(28.28%)
**No ICU admission**	388(71.71%)
**Laboratory indicators**	
Hemoglobin (gm/dL)	13.22±2.04
WCC (x10(3)/mcL)	8.65±7.80
Neutrophil count (x10(3)/mcL)	7.85±35.53
Lymphocyte count (x10(3)/mcL)	1.36±0.73
Platelet count (x10(3)/mcL)	249.4±99.56
INR	1.05±1.16
D-dimer (mg/l)	2.35±5.64
Ferritin (mcg/L)	929.6±1363
CRP (mg/l)	81.45±96.54
Urea (mmol/L)	7.40±26.40
Creatinine (μmol/L)	116.9±312.5
Sodium (Na) (mmol/L)	136.2±4.677
Potassium (K)(mmol/L)	4.13±1.95
LDH (IU/L)	415.4±319.3
Serum bilirubin (μmol/L)	15.77±38.70
ALT (IU/L)	67.49±118.7
AST (IU/L)	58.73±114.7
ALP (IU/L)	88.02±53.70
Albumin(gm/L)	30.83±7.193
Procalcitonin (μg/L)	0.88±4.18

SD; standard deviation, No; number, %; percentage, BMI; body mass index, ICU; intensive care admission, WCC; white cell account, INR; international normalized ratio, CRP; C-reactive protein, LDH; lactate dehydrogenase, ALT; alanine aminotransferase, AST; aspartate transaminase, ALP; alkaline phposphatase

### Elevated inflammatory markers are associated with worse clinical course in patients with COVID‐19 infection

As shown in Table 2, we investigated the association between inflammatory markers and COVID‐19 clinical severity. Our results showed a significant association between inflammatory markers and the clinical outcome. WBC count was significantly elevated in the critical COVID-19 patients (10.0±4.73 10^3^ cells/μL) compared to 8.92±11.50 10^3^ cells/μL in the severe and only 7.30±3.41 10^3^ cells/μL in patients who suffer from mild form of the COVID-19 infection. Similarly, neutrophils count, CRP, D-Dimer, ferritin PCT, and LDH levels were all significantly higher in patients suffering from the severe and critical forms compared to the mild to moderate form of the disease. Patients that needs ICU admission also showed significantly higher levels of WBC count, neutrophils count, CRP, D-dimer, ferritin, procalcitonin and LDH. Moreover, those patients showed significantly lower lymphocyte count (<0.0001). Similar picture was also observed with patients that need ventilation compared to patients with no need of ventilation and patients that suffers from ARDS compared to patients with no evidence of ARDS. Indeed, deceased patients also showed significant higher levels of WBC count, neutrophils count, CRP, D-dimer, ferritin, procalcitonin, LDH as well as lower lymphocyte count compared to patients that stay alive.

**Table 2:** The association between inflammatory markers and clinical behaviour of COVID19 patient’s.

	WBC count	Neutrophils count	Lymphocytes count	CRP	D-Dimer	Ferritin	Procalcitonin	LDH
**Disease severity**								
Mild-moderate	7.30±3.41	4.89±3.18	1.75±0.74	20.59±34.78	0.95±2.28	330.8±445.4	0.31±1.69	268.7±287.60
Sever	8.92±11.50	6.20±3.28	1.28±0.68	89.65±100.3	1.34±2.7	1100±1053	0.66±4.50	418.1±186.90
Critical	10.0±4.73	13.87±67.29	0.965±0.50	147.5±97.56	5.5±9.20	1582±2001	1.69±5.08	569.5±409.70
P value	0.0056	0.0488	<0.0001	<0.0001	<0.0001	<0.0001	0.0203	<0.0001
**ICU admission**	9.85±4.83	13.54±66.44	0.99±0.48	138.9±99.00	5.06±8.79	1513±1959	1.76±5.33	553.4±410.7
**No ICU admission**	8.18±8.65	5.61±3.24	1.50±0.76	58.80±85.66	1.42±3.27	797±986	0.46±3.43	355.9±248.5
P value	0.0249	0.0193	<0.0001	<0.0001	<0.0001	<0.0001	0.0017	<0.0001
**Died**	10.47±4.56	8.98±3.49	0.89±0.42	138.9±99	5.06±8.79	1493±1954	2.30±6.23	618.4±484.70
**Alive**	8.27±8.27	5.78±4.35	1.45±0.74	58.80±85.66	1.44±3.28	802±987	0.52±3.39	369.5±246.8
P value	0.0133	<0.0001	<0.0001	<0.0001	<0.0001	<0.0001	0.0002	<0.0001
**No Ventilation**	8.20±8.29	5.69±3.32	1.46±0.74	65.95±88.57	1.58±3.88	874.6±1083	0.50±3.72	341.70±232.8
**Ventilation**	10.55±4.88	9.02±4.65	0.92±0.46	145±101	6.22±9.45	1556±2124	1.66±4.96	581.40±413
P value	0.0055	<0.0001	<0.0001	<0.0001	<0.0001	<0.0001	0.0051	<0.0001
**ARDS**	9.91±4.67	8.28±4.370	0.96±0.50	146.5±96.80	5.59±9.15	1571±1962	1.66±4.96	581.4±413.0
**NO ARDS**	7.73±3.52	5.54±3.30	1.52±0.75	54.85±82.9	1.10±2.24	762±931	0.50±3.70	341.7±232.8
P value	<0.0001	<0.0001	<0.0001	<0.0001	<0.0001	<0.0001	0.0050	<0.0001

Data presented as mean ± standard deviation, WCC; white cell count (x10(3)/mcl), neutrophils count, (x10(3)/mcl), lymphocytes count, (x10(3)/mcl), CRP; C-reactive protein (mg/L), D-dimer (mg/l), Ferritin (mcg/L), Procalcitonin (ug/L), LDH; lactate dehydrogenase (IU/L), ARDS, acute respiratory distress syndrome, ICU; intensive care unit.

Students t-test was used to evaluate if the means of two groups in our data were significantly different, whereas, ANOVA test was used if we have three or more groups.

### Low lymphocyte count at admission time is associated with more aggressive clinical COVID-19 disease

Interestingly and among the different inflammatory markers, the absolute lymphocyte count (ALC) showed an opposite trend of association with COVID-19 disease severity. As can be seen in Table 2, patients with lower absolute lymphocyte count were significantly associated with severe to critical COVID-19 diseases compared to the mild to moderate form that showed higher lymphocyte count (P<0.0001). Moreover, patients who presented with features of ARDS showed a significantly lower level of ALC (0.96±0.50× 10^3^ cells/μL) compared to 1.52±0.75× 10^3^ cells/μL in patients with no features of ARDS (P<0.0001). This was reflected on patients who needed ventilation. Indeed, patients who needed ventilation showed a significantly lower levels of ALC (0.92±0.46× 10^3^ cells/μL) compared to patients who did not need ventilation (1.46±0.74× 10^3^ cells/μL) (P<0.0001). Similarly, patients require ICU admission showed significantly lower ALC count (0.99±0.48× 10^3^ cells/μL) compared to 1.50±0.76 10^3^ cells/μL (P<0.0001). The association between ALC count and patient outcome was evident in the strong association between lower ALC count and patient’s death. Patients who died from complications of COVID-19 infection showed a significantly lower ALC count (0.89±0.42× 10^3^ cells/μL) compared to patients who survived (1.45±0.74× 10^3^ cells/μL) (P<0.0001).

#### Elevated inflammatory markers correlate with multiple organ dysfunction including liver, renal and cardiac injuries

As can be seen in Table 3, our results showed a significant association between a group of inflammatory markers and different organs dysfunctions including liver, renal cardiac injuries. Elevated white blood cells (WBC) count; P = 0.0241, neutrophils count (P<0.0001); CRP (P<0.0001); D-Dimer (P<0.0001); serum ferritin (P = 0.0077); PCT (P = 0.0005) and LDH levels were significantly associated with acute cardiac injury. Similarly, elevated WBC count; (P = 0.0020), neutrophils count (P<0.0001); CRP (P<0.0001); D-Dimer (P<0.0001); serum ferritin (P<0.0001); PCT (P<0.0001) and LDH levels (P = 0.0002) showed a significant association with acute renal injury. In contrast, only elevated neutrophils count (P = 0.0007); CRP (P<0.0001); serum ferritin (P<0.0001) and LDH levels (P<0.0001) showed a significant association with acute liver injury.

**Table 3:** The association between inflammatory markers and organs injury among 541 symptomatic COVID-19 patients.

	WBC count	Neutrophils count	Lymphocytes count	CRP	D-Dimer	Ferritin	Procalcitonin	LDH
**Acute cardiac injury**	10.27±4.55	8.61±4.50	1.08±0.628	132.6±105.6	5.96±9.4	1318±1891	2.10±5.91	557.7±449.7
**No Acute cardiac injury**	8.38±8.97	5.88±3.41	1.38±0.72	73.22±90.61	1.43±3.28	932±1171	0.489±3.39	379.1±248.5
P value	0.0241	<0.0001	<0.0001	<0.0001	<0.0001	0.0077	0.0005	<0.0001
**Acute renal injury**	10.68±4.92	8.97±4.69	0.99±0.52	131±94.74	5.19±8.01	1504±2099	2.194±6.10	572.4±471.1
**No Acute renal injury**	8.13±8.31	5.65±3.27	1.456±0.75	68.51±92.85	1.81±4.80	875±1069	0.494±3.32	371.1±244.2
P value	0.0020	<0.0001	<0.0001	<0.0001	<0.0001	<0.0001	<0.0001	0.0002
**Acute liver injury**	9.21±3.97	7.49±3.83	1.107±0.56	126±131	3.03±5.88	1781±2171	1.20±5.34	567.7±485.8
**No Acute liver injury**	8.52±8.45	6.06±3.80	1.42±0.75	70.98±83.27	2.32±5.55	813±1015	0.82±3.86	377.3±248.2
P value	0.4192	0.0007	<0.0001	<0.0001	0.2602	<0.0001	0.4287	<0.0001

WBC; white blood cells count (x10(3)/mcl), neutrophils count, (x10(3)/mcl), lymphocytes count, (x10(3)/mcl), CRP; C-reactive protein (mg/L), D-dimer (mg/l), Ferritin (mcg/L), procalcitonin (ug/L) LDH; lactate dehydrogenase (IU/L). Data presented as mean ± standard deviation, Students t-test was used to evaluate if the means of two groups in our data were significantly different.

#### Low absolute lymphocyte count (ALC) at admission time is associated with multiple organ dysfunction

Our results also highlighted a significant association between lymphopenia and multiple organ injury. Indeed, patients with evidence of acute cardiac injury showed a significantly lower ALC (1.08±0.628× 10^3^ cells/μL) compared to (1.38±0.72× 10^3^ cells/μL) (P<0.0001) (Table 3). Similarly, patients suffer from acute renal injury also showed significantly lower ALC (1.456±0.75× 10^3^ cells/μL) compared with patients with no evidence of acute renal injury (0.99±0.52× 10^3^ cells/μL) (P<0.0001). In addition, patients with acute liver injury showed lower ALC (1.42±0.75× 10^3^ cells/μL) compared to (1.107±0.56× 10^3^ cells/μL) in patients with no liver injury.

## Discussion

Here we investigated the association between key inflammatory markers and a wide range of clinical characteristics and laboratory variables as well as patient outcome, in a large COVID-19 patients’ cohort. Our results showed elevated CRP, D-Dimer, serum ferritin as well as LDH to have the highest level of significance (P<0.0001) with more severe clinical course including patients with severe and critical forms as well as admission to the ICU unit. Similarly, elevated neutrophils count, in addition to CRP, D-Dimer, serum ferritin as well as LDH were strongly associated with poor outcome presenting as death compared to patients who were alive. This finding goes with recent reports that showed high levels of CRP, PCT, D-dimer, LDH and ferritin are associated with more severe clinical course and are able to predict poor prognosis [23–27].

Many reports observed a significant increment in CRP levels in COVID-19 patients compared to normal individuals, furthermore, higher CRP levels were found in the majority of COVID‐19 patients presented with severe illness compared to mild or non‐severe patients [2, 10, 24, 28–30].

The fact that CRP, is an acute-phase protein, usually used as a marker of systemic inflammation [20, 31, 32], clearly highlighted the possible use of this marker as an early and simple marker to predict the risk of disease progression in COVID‐19 patients [32]. The elevation in CRP levels was closely linked to inflammatory cytokines overproduction as well as tissue destruction seen in patients with severe COVID‐19 illness. For that reason, measuring of pro-inflammatory cytokines including IL-6 in patients serum was also proposed as a potential biomarker not only for risk assessment, but also to monitor disease progression as well as prediction of response to treatment [33].

The D-Dimer, which is an important product of fibrin fibrinolytic degradation that is usually elevated in hypercoagulable state, usually used to evaluate deep vein thrombosis or pulmonary embolism as well as risk of abnormal blood clotting [34] as well as the presence of disseminated intravascular coagulation [35, 36]. Previous reports clearly linked between elevated levels of D-dimer and disease progression in COVID-19 patients. Indeed, patients that need ICU admission showed a significantly higher D-dimer levels. Similarly, elevated levels of D-dimer were also observed in patient with severe illness and strongly correlated with higher mortality [2, 37]. Our lab also observed that among several clinco-pathological characteristics and biochemical markers, D-dimer was among three predictors that can identify patients with higher risk to develop critical COVID-19 illness [6].

Procalcitonin (PCT), which is the precursor of calcitonin and synthesised by thyroid parafollicular C cells, was found to be synthesized during bacterial infection in several extrathyroid tissues. This process is mediated by tumour necrosis factor-alpha (TNFα) and interleukin 6 activity [38].Many reports also highlighted a strong association between elevated PCT and severe COVID-19 [39–41].

While the LDH, which is widely distributed intracellular enzyme that play an essential role in carbohydrate metabolism through catalyzing interconversion of lactate and pyruvate with concomitant interconversion of NAD+/NADH coenzyme system was not considered as a classical marker of inflammation, severe infections were found to provoke cytokine-mediated tissue damage as well as LDH release [42]. This increment in LDH level was considered as an indicator of tissue and cell destruction as well as damage usually induced by SARS-CoV-2 [43]. For that reason, elevated LDH levels also was considered as a predictor of progression to sever illness and higher mortality rates [21, 23, 43–45].

Another important finding in our study is the strong association between ALC and more severe disease and worse outcome including the need for ventilation, ICU admission and higher mortality rate. Lymphopenia was previously reported in a group of viral infections including SARS and Middle East Respiratory Syndrome (MERS) [46–48]. Also, several reports showed a significant association between lower ALC and severe COVID 19 illness [49–51]. In addition, other report showed that COVID-19 survivors were found to have significantly higher lymphocyte count compared to non-survivors [18, 50]. Moreover, our finding that lower ALC to be associated with multiple organ injury including acute liver and renal injuries go with other reports that showed patients who developed acute kidney injury were more likely to have lymphocytopenia compared with patients without acute kidney injury [52]. While the mechanism that might explain the association between lymphocytopenia and poor patients’ outcome in COVID-19 patients is not fully understood, previous reports in other similar beta-CoV infections including (SARS)-CoV and (MERS)- CoV highlighted the possible role of lymphocyte sequestration in specific target organs which might lead to rapid reduction in both CD4+ and CD8+ T lymphocytes [53]. Other proposed mechanisms for this reduction include the fact that the angiotensin coverting enzyme-2 (ACE2) receptor, which is essential for COVID-19 pathogenesis is also expressed in lymphocytes, which might render them a direct target of SARS-CoV-2 infection [54]. Moreover, the increment of pro-inflammatory cytokines including interlukin-6 (IL-6) that is usually observed in COVID-19 might lead to further lymphocyte reduction [55].

Finally, our observation that inflammatory markers are significantly associated with organs dysfunctions including liver, renal cardiac injuries raise the question, if these dysfunctions are a result of COVID-19 tissue destruction or it is related with the comorbidity usually associated with the severe disease. Further studies are need to investigate this point.

In conclusion, our results highlighted the significant association between different classical inflammatory markers and clinical as well as laboratory profile of patients with COVID-19 infection. In addition, our results highlighted the possible use of these markers at the time of admission as a potential predictive markers for more severe clinical course in COVID-19 patients which might require more aggressive therapeutic approach including the need of ventilators and ICU admission.
